# Molecular Surveillance and Whole Genomic Characterization of Bovine Rotavirus A G6P[1] Reveals Interspecies Reassortment with Human and Feline Strains in China

**DOI:** 10.3390/vetsci12080742

**Published:** 2025-08-07

**Authors:** Ahmed H. Ghonaim, Mingkai Lei, Yang Zeng, Qian Xu, Bo Hong, Dongfan Li, Zhengxin Yang, Jiaru Zhou, Changcheng Liu, Qigai He, Yufei Zhang, Wentao Li

**Affiliations:** 1 National Key Laboratory of Agricultural Microbiology, College of Veterinary Medicine, Huazhong Agricultural University, Wuhan 430070, China; a.ghonaim@webmail.hzau.edu.cn (A.H.G.); mklei@webmail.hzau.edu.cn (M.L.); zy0802@webmail.hzau.edu.cn (Y.Z.); xq1@webmail.hzau.edu.cn (Q.X.); hong1741@webmail.hzau.edu.cn (B.H.); lidongfan@webmail.hzau.edu.cn (D.L.); silverxin@webmail.hzau.edu.cn (Z.Y.); jiaru@webmail.hzau.edu.cn (J.Z.); changchengliu0813@163.com (C.L.); he628@mail.hzau.edu.cn (Q.H.); 2 Key Laboratory of Preventive Veterinary Medicine in Hubei Province, The Cooperative Innovation Centre for Sustainable Pig Production, Wuhan 430070, China; 3 Desert Research Centre, Cairo 11435, Egypt; 4 Frontiers Science Center for Animal Breeding and Sustainable Production, Wuhan 430070, China; 5 College of Veterinary Medicine, Inner Mongolia Agricultural University, Hohhot 010000, China; 6 Key Laboratory of Clinical Diagnosis and Treatment Technology in Animal Disease, Ministry of Agriculture, Hohhot 010000, China

**Keywords:** bovine rotavirus A, neonatal calf diarrhea, virus isolation, whole-genome sequencing, phylogenetic analysis, cross-species transmission

## Abstract

Group A rotavirus (RVA) is a leading causative agent of diarrhea in both young animals and humans. In China, multiple genotypes are commonly found within the bovine population. In this study, we investigated 1917 fecal samples from calves with diarrhea between 2022 and 2025, with 695 testing positive for RVA, yielding an overall detection rate of 36.25%. The highest positivity rate was observed in Hohhot (38.98%), and annual detection rates ranged from 26.75% in 2022 to 42.22% in 2025. A bovine rotavirus (BRV) strain, designated 0205HG, was successfully isolated from a fecal sample of a newborn calf. Its presence was confirmed through cytopathic effects (CPEs), the indirect immunofluorescence assay (IFA), electron microscopy (EM), and high-throughput sequencing. Genomic characterization identified the strain as having the G6-P[1]-I2-R2-C2-M2-A3-N2-T6-E2-H3 genotype constellation. The structural proteins VP2 and VP7, along with nonstructural genes NSP1–NSP4, shared high sequence identity with Chinese bovine strains, whereas VP1, VP4, and NSP5 clustered more closely with human rotaviruses, and VP3 was related to feline strains. These findings highlight the genetic diversity and interspecies reassortment of BRVs in China, underlining the importance of continued surveillance and evolutionary analysis.

## Simple Summary

Rotavirus A (RVA) is a primary pathogen that induces diarrhea in both animals and humans. In this study, we analyzed nearly 2000 fecal samples collected from diarrheic calves in China between 2022 and 2025. We found that over one-third of the samples tested positive for RVA, with the highest infection rate recorded in Hohhot. From these, we successfully isolated a bovine RVA strain named 0205HG. Whole-genome analysis revealed that while most of its genes were closely related to bovine RVA strains in China, some segments were genetically similar to human and feline rotaviruses. This indicates that different species may share or exchange rotavirus genes. Our findings underscore the necessity for ongoing monitoring of RVA in animals, as such cross-species transmission could pose a risk to animal and human health.

## 1. Introduction

Neonatal calf diarrhea is a major cause of morbidity and mortality in young calves, responsible for over half of all calf mortalities [1,2]. This condition imposes significant economic burdens and reduces productivity within the cattle industry [3]. The causes of calf diarrhea are diverse and include viruses, bacteria, and protozoa. Among the primary pathogens involved are bovine group A rotavirus (BRVA), bovine coronavirus (BCoV), *Clostridium perfringens* type C, *Cryptosporidium parvum*, *Salmonella* species, and *Escherichia coli* [4]. It is important to note that many of these enteropathogens can be detected in healthy calves without causing disease, indicating that their presence alone does not confirm causation [5]. Often, calf diarrhea results from multiple pathogens acting in concert rather than a single infectious agent.

Rotavirus (RV) is a primary etiological factor of acute diarrhea in calves under one month of age. Additionally, it is a prevalent etiological factor of diarrheal diseases in young infants and various animal species globally, with notably high incidence rates documented in developing regions of Africa and Asia [6,7]. Among the pathogens responsible for neonatal calf diarrhea, the majority of cases are attributed to BRVA, bovine coronavirus (BCoV), and bovine norovirus, while approximately 8% of cases involve other agents [8]. Calves between birth and four weeks of age are particularly vulnerable due to their underdeveloped immune systems, and maintaining calf health during this critical window is essential for the long-term sustainability of both dairy and beef operations [4]. As calves approach the weaning stage, strengthening their immune defenses and minimizing environmental exposure to infectious agents becomes increasingly important [9,10]. Despite advancements in diagnostics, veterinary care, and management practices, calf morbidity and mortality remain persistent challenges for livestock producers globally. Notably, these issues continue to pose significant concerns even in high-income countries, where well-resourced production systems have implemented modern health and husbandry protocols [1,11].

Understanding the risk factors that influence neonatal calf health is essential for developing effective prevention and control strategies [12,13]. However, studies have indicated that widespread vaccination efforts may exert selective immune pressure on circulating rotavirus strains, potentially driving viral evolution and the emergence of novel genotypes [3,7]. This antigenic variation may reduce the efficacy of existing vaccines and complicate disease control. The prevalence of BRV-induced diarrhea varies substantially across countries, ranging from 7% to 94%, with a reported rate of approximately 46% in China [14]. Notably, calves under 7 days of age are particularly susceptible to severe infection and clinical disease [15].

The BRV genome consists of eleven segments of double-stranded RNA, encoding eleven proteins, including six structural and five nonstructural proteins. Rotavirus classification has traditionally relied on the VP7 and VP4 genes. This dual typing system has evolved into a full-genome classification approach, involving nucleotide sequencing of all 11 segments and assigning segment-specific identity cutoffs. In this system, the segments are denoted as follows: VP7-VP4-VP6-VP1-VP2-VP3-NSP1-NSP2-NSP3-NSP4-NSP5/6, labeled Gx-P[x]-Ix-Rx-Cx-Mx-Ax-Nx-Tx-Ex-Hx, respectively [16,17].

Due to frequent point mutations, BRV gene segments can give rise to novel strains, with the potential for interspecies transmission and an expanded host range [18]. BRV strains are categorized by antigenic typing of the VP4 and VP7 proteins into five G genotypes (G1, G6, G8, G10, G15) and three P genotypes (P1, P5, P11) [5]. In China, the predominant BRV genotypes are G6P[1], G6P[5], and G10P[11] [14]. Diarrhea in calves can be reduced by implementing suitable management practices and vaccination strategies for pregnant cows. However, there are several reasons why vaccines may fail, such as genetic reassortment between different strains, variations in antigenic or genetic characteristics, interactions among various or less common genotypes, lack of sufficient heterologous immunity, and poor vaccination management [19,20,21]. A study has indicated that cross-protection among various rotavirus genotypes is relatively ineffective, with reported protection rates ranging from 23% to 85% [22].

For successful control and prevention of BRV infections, it is essential to understand the virus’s epidemiological trends and potential for zoonotic transmission. Therefore, examining the genetic diversity and reassortment dynamics of RV strains is crucial for improving disease control efforts.

In this study, fecal samples were obtained from calves raised on farms in Shandong Province and the Inner Mongolia Autonomous Region of China. The BRV strain present in these samples was isolated and genetically characterized. Full-genome sequencing was performed, followed by phylogenetic analysis. The insights gained from this study will enhance our understanding of the evolutionary relationships among BRV strains and support the development of more effective vaccines and control strategies.

## 2. Materials and Methods

### 2.1. Clinical Sample Collection and Preparation

A total of 1917 samples, comprising fecal matter and anal swabs, were obtained from calves under four weeks of age exhibiting diarrhea on various cattle farms located in Shandong Province and the Inner Mongolia Autonomous Region between 2022 and 2025. All sampled calves were clinically diagnosed with diarrhea, and all were offspring of dams that had not been vaccinated against rotavirus. More detailed information, such as exact weekly age segmentation, rearing system (intensive vs. free-range), and farm scale, was not available. Samples were transported to the laboratory under dry ice conditions and preserved at −80 °C until they were processed. For sample preparation, each fecal specimen was mixed with high-glucose Dulbecco’s Modified Eagle Medium (DMEM; Gibco, Waltham, MA, USA) at a 1:10 ratio. The mixture was then subjected to centrifugation at 300× *g* for 10 min. The resulting supernatant underwent further clarification through a second centrifugation at 8000× *g* for 30 min. The final supernatants were stored again at −80 °C for subsequent RNA extraction and viral isolation. All sampling and laboratory work were carried out between 2022 and 2025.

### 2.2. Genomic Screening for Rotavirus Group A

Fecal samples were prepared by diluting them at a 1:9 (*w*/*v*) ratio in sterile phosphate-buffered saline (PBS). The mixtures were then centrifuged at 16,000× *g* for 10 min using a Thermo Fisher Heraeus Multifuge X1R centrifuge (Thermo Fisher Scientific, Waltham, MA, USA) to obtain clear supernatants. Viral RNA was isolated from these supernatants utilizing the RNA Fast 200 Total RNA Extraction Kit (Feijie Biotek, Inc., Shanghai, China), following the protocol provided by the manufacturer. The RNA yield was measured with a NanoVue Plus spectrophotometer (Thermo Fisher Scientific, Waltham, MA, USA), and RNA purity was determined by measuring the A260/280 absorbance ratio. Complementary DNA (cDNA) was synthesized using the High-Capacity cDNA Reverse Transcription Kit (Applied Biosystems, Waltham, MA, USA). RVA detection was performed using reverse transcription PCR (RT-PCR) following a previously published protocol [23], employing primers VP6-F (5′-GGCTTTWAAACGAAGTCTTC-3′) and VP6-R (5′-GGYGTCATATTYGGTGG-3′). PCR reactions were conducted in a total volume of 50 µL. The amplified DNA fragments were subjected to agarose gel electrophoresis and subsequently visualized under UV light following ethidium bromide staining.

### 2.3. Virus Isolation Workflow

Fecal specimens that tested positive for BRV were chosen for virus isolation based on previously reported protocols [24]. The supernatants from these samples were passed through 0.45 µm polytetrafluoroethylene (PTFE) syringe filters for further processing. To enhance viral infectivity, bovine trypsin (Sigma-Aldrich, St. Louis, MO, USA) was added to the filtrates at a concentration of 10 µg/mL and incubated for 1 h. Prior to inoculation, cell monolayers were washed thrice with prewarmed, serum-free DMEM. Trypsin-treated samples were inoculated onto cells and incubated for 60 to 90 min at 37 °C in a humidified atmosphere containing 5% CO_2_. After this adsorption period, the inoculum was discarded, and the cells were rinsed with prewarmed serum-free DMEM before continuing incubation under the same conditions. Upon the appearance of CPEs, the culture supernatant containing the virus was harvested for subsequent serial passaging.

To further characterize the isolated BRV strain, the immunofluorescence assay (IFA) and transmission electron microscopy (TEM) were employed [25]. For the IFA, MA-104 cells were infected with the BRV isolate at a multiplicity of infection (MOI) of 0.1 and cultured for 36 h. Post incubation, the cells were fixed with 4% paraformaldehyde and then blocked with 5% skim milk at room temperature for 2 h. The cells were subsequently incubated for 1 h at 37 °C with a primary monoclonal antibody specific to BRV VP6 (1:500 dilution), produced and maintained in our laboratory. Secondary detection was performed using Alexa Fluor 594-conjugated goat anti-mouse IgG (H + L) antibodies (Sino Biological, Beijing, China). Fluorescent signals were then visualized under a fluorescence microscope.

For TEM analysis, viral suspensions were concentrated by centrifugation at 30,000× *g* for 30 min. The resulting viral pellet was subjected to negative staining with 2% phosphotungstic acid (pH 7.0) following an established protocol and imaged using a Hitachi 7650 transmission electron microscope (Hitachi High-Technologies Corporation, Tokyo, Japan). The isolated strain was identified via RT-PCR, and the BRV isolate was designated 0205HG.

### 2.4. Viral Plaque Purification and Growth Curve Determination

The BRV strain 0205HG was isolated and purified by performing plaque cloning assays using MA104 cell cultures. Initially, both viral strains were treated with 10 µg/mL trypsin for 1 h. Serial dilutions of the 0205HG strain were then prepared and used to infect MA104 cells in post-inoculation medium, followed by a 90 min incubation at 37 °C in a 5% CO_2_ environment. After the adsorption period, the inoculum was carefully removed, and each well was overlaid with 2 mL of post-inoculation medium containing 1.5% agarose to facilitate plaque formation. Once the overlay solidified, the plates were further incubated at 37 °C in a 5% CO_2_ environment to facilitate plaque formation. Visible plaques were collected using a micropipette, resuspended in 1 mL of DMEM, and subjected to three cycles of freezing and thawing. The resulting lysates were used to infect fresh MA104 cells for subsequent rounds of plaque purification. This procedure was repeated three times. After 48 h of incubation, the agarose overlay was removed. Once plaques reached the optimal size, the cells were fixed with 4% paraformaldehyde for 2 h, and plaque visualization was performed.

To assess viral replication, the growth curve of strain 0205HG in MA104 cells was generated based on the 50% Tissue Culture Infectious Dose (TCID_50_). MA104 cells were seeded in 96-well plates at a density of 1 × 10^5^ cells per well in 100 μL of culture medium and incubated for 48 h at 37 °C in a humidified atmosphere containing 5% CO_2_. After discarding the culture medium, 100 µL of the virus, prepared in tenfold serial dilutions, was inoculated into each well. The cells were then monitored every 12 h for cytopathic effects (CPEs) over a period of seven days following inoculation to assess viral replication and pathogenicity. Viral titers were calculated using the TCID_50_ assay according to the Karber method.

### 2.5. Whole-Genome Sequencing

To obtain the full-genome sequence of the detected RVA, next-generation sequencing (NGS) was performed on a fecal sample that showed strong positivity by RT-PCR. Sequencing was carried out on the Illumina MiSeq platform using the V2 sequencing kit, which produced 250-base-pair paired-end reads, along with the NuGen Trio RNA library preparation kit. The resulting FASTQ files were analyzed using a custom in-house bioinformatics pipeline. Trimmomatic (v0.39) was employed for trimming to eliminate Illumina adapters, and a minimum quality score threshold of 20 was applied [26]. Subsequently, Bowtie 2 (v2.4.4) was utilized to remove any host contamination [27]. The remaining unmapped reads were assembled using SPAdes (v3.15.2) with k-mer sizes of 21, 31, 41, 51, 61, and 71 and the “careful” option enabled [28]. Resulting contigs were taxonomically classified using BLASTx against the NCBI database.

### 2.6. Viral Segment Genotyping and Phylogenetic Analysis

The genotyping of all complete viral genome segments was conducted using the Rotavirus A Genotype Determination Tool provided by the Virus Pathogen Database and Analysis Resource (ViPR) [29]. Relevant sequences matching the targeted genomic segments were retrieved from the NCBI nucleotide database (Supplementary Table S1) and subjected to multiple sequence alignment using the ClustalX v1.83 algorithm. Subsequently, phylogenetic trees for the whole-genome sequences were generated via Molecular Evolutionary Genetics Analysis (MEGA) software version 6.06, employing the neighbor-joining method with 1000 bootstrap replicates [30]. Trees were exported in Newick format and visualized with iTOL (https://itol.embl.de (accessed on 1 June 2025), which allowed for interactive annotation and color-coding [31].

## 3. Results

### 3.1. Positive Rate of Bovine Rotavirus A

**Figure 1:**
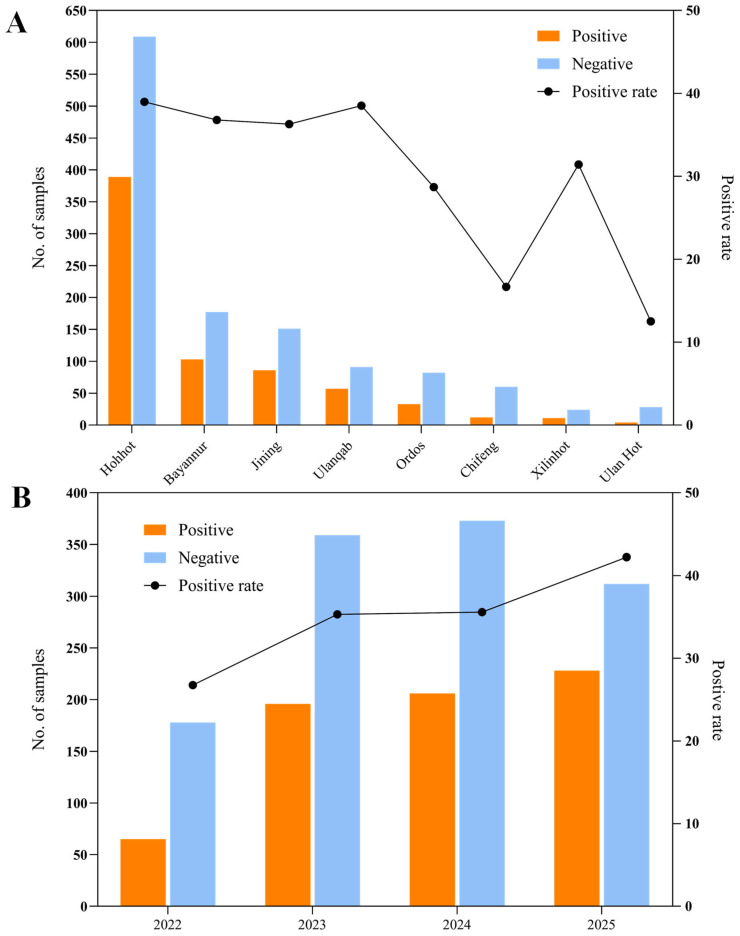
Geographic and temporal distribution of bovine rotavirus (BRV) cases in China. (**A**) Spatial distribution of BRV-positive and BRV-negative samples across various Chinese cities. (**B**) Yearly trends in BRV positivity rates from 2022 to 2025, illustrating temporal changes in detection rates over the study period.

Out of the 1917 calf samples analyzed, 695 tested positive for rotavirus, resulting in an overall detection rate of 36.25% (Figure 1A). The data indicated notable differences in prevalence across various cities, with positivity rates ranging from 12.5% to 38.98%. On average, the detection rate stood at 36.25%. Among the surveyed areas, Hohhot reported the highest rate of bovine rotavirus A (BRVA) positivity at 38.98%, surpassing those observed in the other locations. To account for potential temporal variations, BRV-positive rates were analyzed across the study period (2022–2025), revealing fluctuations in annual detection rates, as illustrated in Figure 1B, with 2022 showing the lowest positive rate (26.75%) and 2025 showing the highest positive rate (42.22%).

### 3.2. Viral Isolation and Identification

**Figure 2:**
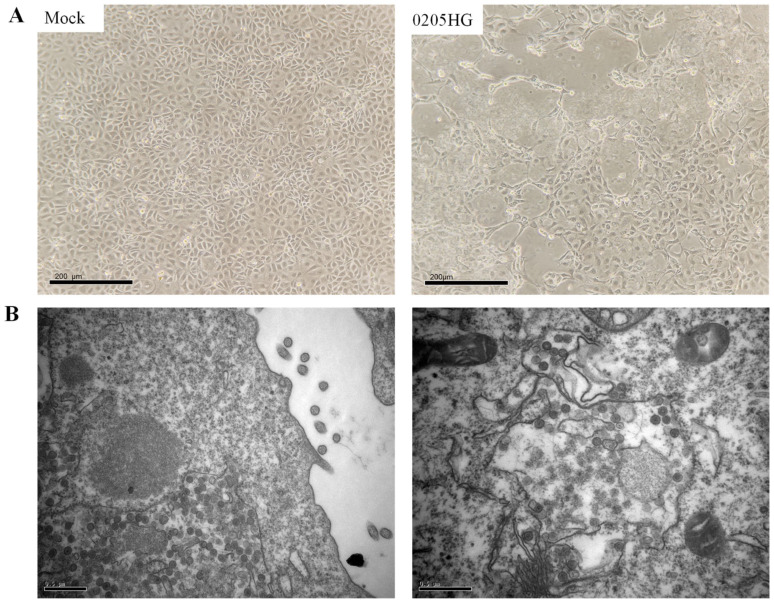
Characteristics of the 0205HG isolate cultured from MA104 cells. (**A**) CPEs induced by 0502HG in MA104 cells. (**B**) Electron microscopy images of purified BRV particles.

All RT-PCR-positive samples were subjected to virus isolation using the MA104 cell line. Prior to inoculation, samples were treated with trypsin to enhance viral infectivity. Although multiple positive samples were tested, successful propagation in MA104 cells was achieved with only one isolate, designated 0205HG. This limited success is likely due to the inherently low efficiency of rotavirus isolation from field samples. Cytopathic effects (CPEs) became apparent by the third viral passage. At 24 h post infection (p.i.), infected cells began to round up and aggregate. By 72 h p.i., cell shrinkage was evident, and most of the monolayer had detached (Figure 2A). To visualize the viral particles, transmission electron microscopy (TEM) was conducted on MA104 cells infected with the 0205HG isolate. Typical rotavirus particles, which appeared wheel-shaped, were detected in the cell culture medium (Figure 2B). Following centrifugation of the whole-cell lysates, the resulting supernatants were collected and preserved at −80 °C.

### 3.3. Indirect Immunofluorescence Assay

**Figure 3:**
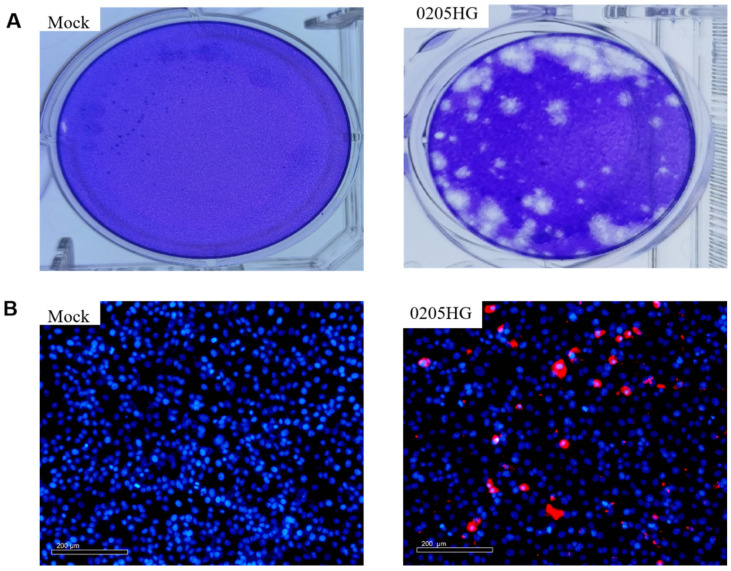
Infection dynamics of the 0205HG isolate in MA104 cells. (**A**) Representative plaques of isolate-infected MA104 cells. Mock and 0205HG-infected MA104 cells at 72 h. (**B**) Red fluorescence was detected in MA104 cells at 12 h post infection with the isolate.

The isolate underwent three rounds of plaque purification (Figure 3A). Additionally, the isolated strain containing the VP6 gene was identified via the IFA. The results indicated that the antibodies selectively bound to the viral proteins, while the control samples presented no specific fluorescence signals (Figure 3B).

### 3.4. TCID_50_ Measurements of the Isolated Strain

**Figure 4:**
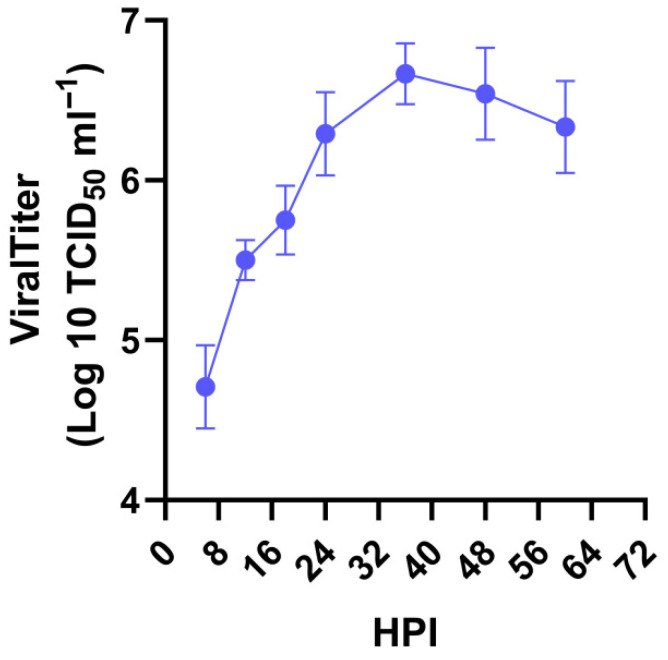
Growth kinetics of 0205HG in MA104 cells over time. MA104 cells were infected with the 0205HG strain at an MOI of 0.01, and cell cultures were collected at the indicated times to determine the viral titers by TCID_50_. The results are the means of three determinations; error bars indicate the standard deviation.

The growth curve of the 0205HG strain was plotted at various time points post infection on the basis of the TCID_50_. The results revealed a rapid increase in the titer from 6 to 36 h post infection (hpi), with virus titers reaching approximately 1 × 10^6.66^ TCID_50_/mL for the 0205HG strain (Figure 4).

### 3.5. Whole-Genome Sequence of the BRV Strain 0205HG

The full genome of the bovine rotavirus (BRV) strain 0205HG, comprising all 11 segments, was successfully sequenced. The resulting sequences have been submitted to the GenBank repository with accession numbers PV579857 to PV579867. The strain was classified with the genotype constellation G6P[1]-I2-R2-C2-M2-A3-N2-T6-E2-H3. Comparative analysis of the nucleotide sequences of the coding regions revealed that six segments (VP2, VP6, NSP1, NSP2, NSP3, and NSP4) shared high similarity with corresponding segments from other BRV strains, with sequence identities of 96.13, 90.51, 99.63, 94.48, 95.49, 98.84, and 99.55%, respectively. Additionally, three segments (VP1, VP4, and NSP5) demonstrated the closest genetic relationship to human rotavirus A (RVA) strains, with sequence identities of 93.4%, 91.8%, and 97.82%, respectively. Notably, the VP3 gene shared the highest sequence identity (90.51%) with a feline rotavirus strain (Table 1).

BLASTn analysis demonstrated that each of the genome segments had nucleotide identities ranging from 90.51% to 99.63% when compared with the most similar sequences in the GenBank database. The genotype constellation of the 0205HG strain, along with reference strains and the closest matches identified in GenBank, is summarized in Table 2.

### 3.6. Molecular Phylogeny of Structural Protein Genes

**Figure 5:**
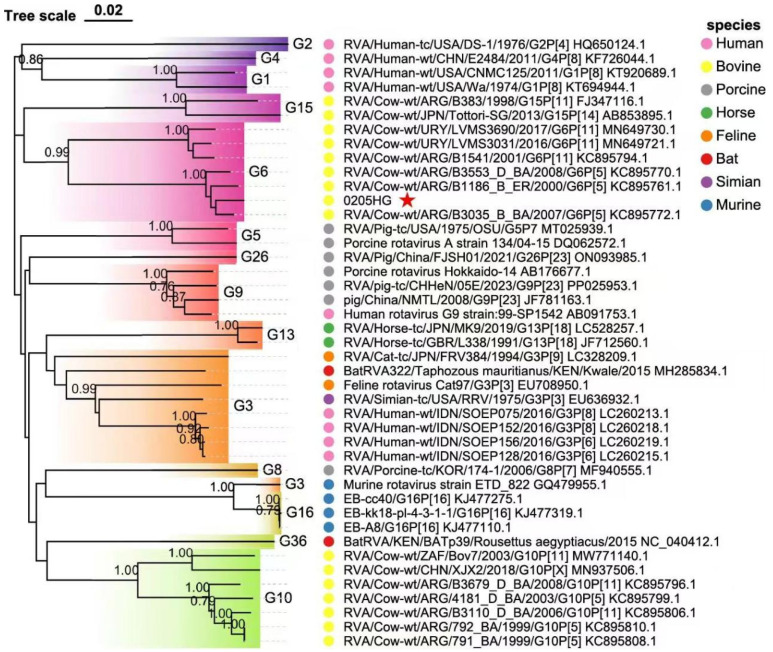
Phylogenetic analysis of the VP7 gene sequence of BRV. The trees were created via neighbor-joining analysis via MEGA 11 software with 1000 bootstrap replicates. The red five-pointed star denotes the VP7 sequence of the 0205HG isolate.

**Figure 6:**
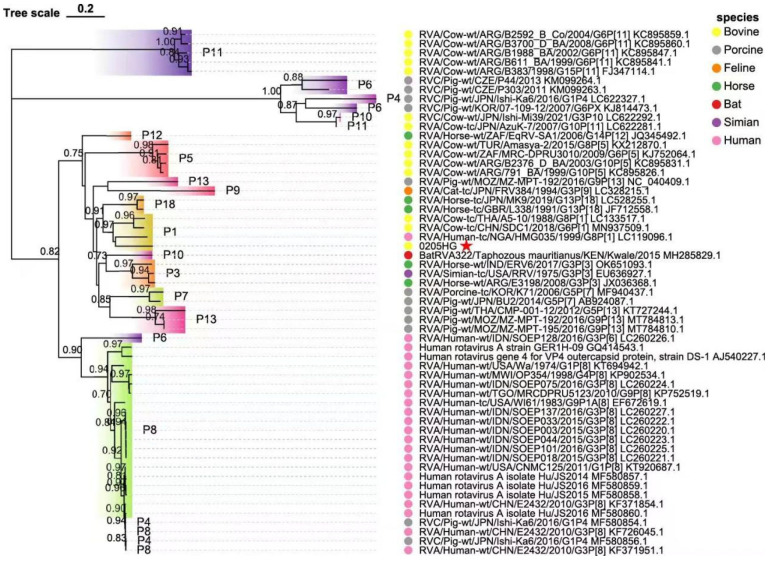
Phylogenetic analysis of the VP4 gene sequence of BRV. The trees were created via neighbor-joining analysis via MEGA 11 software with 1000 bootstrap replicates. The red five-pointed star denotes the VP4 sequence of the 0205HG isolate.

**Figure 7:**
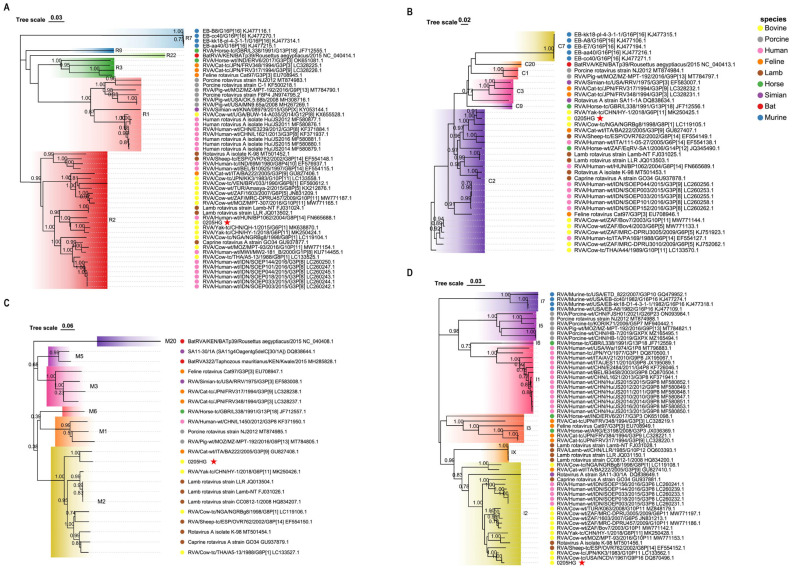
Evolutionary relationships among other BRV genes encoding structural proteins. Panels (**A**–**C**) illustrate the phylogenetic trees for the VP1, VP2, and VP3 genes, respectively, while panel (**D**) depicts the tree for the VP6 gene. Phylogenetic trees were constructed using the neighbor-joining method in MEGA 11 software, with 1000 bootstrap replicates. The red five-pointed star denotes the sequences of the 0205HG isolate.

Phylogenetic analysis of the structural gene segments of the G6P[1] strain revealed that its VP7 and VP4 genes are closely related to various BRV strains with G6 and P[1] genotypes (Figure 5 and Figure 6). The VP7 gene of strain 0205HG clustered closely with the bovine strain RVA/Cow-wt/ARG/B3035_B_BA/2007/G6P[5] (Figure 5). Meanwhile, the VP4 gene was most closely related to the human rotavirus strain RVA/Human-tc/NGA/HMG035/1999/G8P[1] isolated in Nigeria (Figure 6). The VP1 gene of the BRV strain 0205HG clustered with the Hungarian human strain RVA/Human-wt/HUN/BP1062/2004/G8P14 within the R2 genotype. Interestingly, BRV 0205HG was also closely related to RVA/Yak-tc/CHN/QH-1/2015/G6P1 and RVA/Yak-tc/CHN/HY-1/2018/G6P11 (Figure 7A). The VP2 segment shared the closest similarity with that of the bovine yak strain RVA/Yak-tc/CHN/HY-1/2018/G6P[11], both of which belong to the C2 type (Figure 7B). For the VP3 gene, the highest genetic similarity was observed with a feline rotavirus strain, RVA/Cat-wt/ITA/BA222/2005/G3P[9], originating from Italy, both of which belong to the M2 genotype (Figure 7C). The VP6 segment was found to be most closely related to the RVA/Cow-tc/USA/NCDV/1967/G9P16 strain and belongs to the I2 genotype (Figure 7D). Collectively, these findings confirm that the structural genes of the BRV strain 0205HG correspond to the genotypes G6, P[1], I2, R2, C2, and M2.

### 3.7. Molecular Phylogeny of Nonstructural Protein Genes

**Figure 8:**
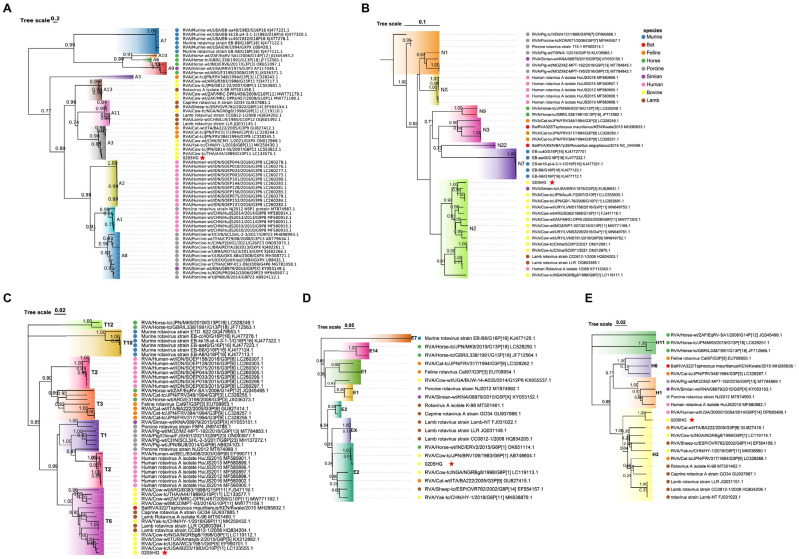
Evolutionary analysis of BRV genes encoding nonstructural proteins. Panels (**A**–**E**) present the phylogenetic trees for the NSP1 to NSP5 genes. Phylogenies were constructed using the neighbor-joining method in MEGA version 11, with 1000 bootstrap replicates. The red five-pointed stars denote the nonstructural protein sequences of the 0205HG isolate.

The NSP1 gene of BRV strain 0205HG shared high genetic similarity to that of the bovine strain RVA/Cow-tc/THA/A44/1989/G10P11, which belongs to the A3 genotype (Figure 8A). The NSP2 segment was most closely related to the corresponding gene in the bovine strain RVA/Cow-tc/JPN/AzuK-7/2007/G10P[11]. Interestingly, NSP2 of 0205HG also shared a close evolutionary relationship with the simian strain RVA/Simian-tc/USA/RRV/1975/G3P[3], with both classified under the N2 genotype (Figure 8B). Additionally, the NSP3 and NSP4 genes of BRV strain 0205HG clustered alongside those of bovine strains RVA/Cow-tc/USA/B223/1983/G10P[11] and RVA/Cow-tc/JPN/BRV106/1983/G6P[1], which belong to the T6 and E2 genotypes (Figure 8C,D). In contrast, the NSP5 gene was most closely related to a human rotavirus strain, RVA/Human-wt/USA/3000015004/2014/G6P[14], which belongs to the E2 genotype (Figure 8E). These phylogenetic findings demonstrate that the nonstructural gene segments of BRV strain 0205HG fall into the genotypes A3, N2, T6, E2, and H3.

## 4. Discussion

Rotaviruses (RVs) are major causative agents of diarrhea in calves, and the BRV prevalence differs globally [32,33]. Globally, BRV is recognized as the leading pathogen of acute diarrhea in calves younger than one month. Moreover, it is also regarded as a potential contributor to acute diarrheal illness in other animal species and, in some cases, humans [6,34]. Consequently, BRV has a serious impact on animal health and the safety of food derived from animals while also posing a possible threat to public health. The acquisition and analysis of rotavirus isolates from the environment are essential not only for tracking the epidemiological landscape of specific regions but also for confirming the efficacy of vaccines and immunotherapies in practical applications. In Brazil, RVA was identified in 25.4% (17 out of 67) of fecal samples from diarrheic calves [35]. In a study from India, BRV was identified in 3 of 45 samples (6.66%) collected from necropsied calves [36]. Additionally, research conducted in 2020, which examined fecal samples from calves across 39 cattle farms in northwestern Argentina between 2014 and 2016, revealed BRV infections in 20 of those farms, with a positivity rate of 8.4% (67 out of 795 samples) [37]. These data highlight that BRV continues to be a major etiological agent of neonatal diarrhea in calves across the globe. The infection is associated with high rates of illness and death, resulting in considerable economic losses for the dairy and beef industries due to both direct and indirect effects.

Rotavirus (RV) is widely prevalent globally, with infection rates ranging from approximately 20.00% to 70% in various studies. In China, multiple provinces and regions have experienced outbreaks of calf diarrhea attributed to RV infection [14,38]. Among dairy calves in China, BRV is frequently detected, with the G6P[1] genotype identified as the most dominant strain [39]. Genetic reassortment can promote the transmission of rotaviruses between species, resulting in genetic similarities among those affecting different animals. There have been documented instances of RVA transmission from cattle to other animals, including rabbits (K1130027 strain) and horses (OH-4 strain) [40]. More recent research has proposed the possibility of bovine-to-feline transmission, revealing that the NSP5 gene of a feline RVA strain shared 97% nucleotide identity with that of a BRV strain, suggesting its origin was likely due to interspecies reassortment from bovine rotavirus [21].

This study detected rotavirus in 695 out of 1917 calf samples (36.25%), with significant variation in prevalence across cities (12.5–38.98%), and the highest positive rate was observed in Hohhot (38.98%), which is consistent with previous reports [19,22,34]. Moreover, we successfully isolated a BRV strain with the G6P[1] genotype from fecal samples of diarrheic calves. The full genomic sequence of the isolate, designated 0205HG, was determined. Phylogenetic analysis of all 11 genomic segments indicated that strain 0205HG is a reassortant, comprising gene segments derived from bovine (VP2, VP7, NSP1, NSP2, NSP3, and NSP4), human (VP1, VP4, and NSP5), and feline (VP3) rotaviruses. Despite its genetic relatedness to human strains, the 0205HG strain appears to be distinct from typical human rotavirus genotypes. These findings are consistent with earlier research indicating a complex interplay and frequent reassortment events between animal and human RVA strains. Numerous studies have identified Wa-like genomic constellations (human strain) in various hosts [41]. Porcine genotypes (G3/G4/G5/G11 VP7 and P[6]/P[7] VP4) have been identified in cattle [32]. Moreover, bovine–porcine reassortant RVA strains with beneficial genetic traits have demonstrated the capacity to infect and cause illness in non-native hosts [42,43]. Avian-origin strains, such as G17P[17] and G18P[17], have also been identified in bovine populations. These findings collectively support the occurrence of interspecies transmission from pigs and birds to cattle. Additionally, bovine genotypes VP7 G1, G2, G3, G4, G12, and VP4 P[6] have been found in human RVA cases, highlighting the zoonotic potential of these viruses [6].

This study detected rotavirus in 695 out of 1917 calf samples (36.25%), with significant variation in prevalence across cities (12.5–38.98%), and the highest positive rate was observed in Hohhot (38.98%), which is consistent with previous reports [19,22,34]. Moreover, we successfully isolated a BRV strain with the G6P[1] genotype from fecal samples of diarrheic calves. The full genomic sequence of the isolate, designated 0205HG, was determined. Phylogenetic analysis of all 11 genomic segments indicated that strain 0205HG is a reassortant, comprising gene segments derived from bovine (VP2, VP7, NSP1, NSP2, NSP3, and NSP4), human (VP1, VP4, and NSP5), and feline (VP3) rotaviruses. Despite its genetic relatedness to human strains, the 0205HG strain appears to be distinct from typical human rotavirus genotypes. These findings are consistent with earlier research indicating a complex interplay and frequent reassortment events between animal and human RVA strains. Numerous studies have identified Wa-like genomic constellations (human strain) in various hosts [41]. Porcine genotypes (G3/G4/G5/G11 VP7 and P[6]/P[7] VP4) have been identified in cattle [32]. Moreover, bovine–porcine reassortant RVA strains with beneficial genetic traits have demonstrated the capacity to infect and cause illness in non-native hosts [42,43]. Avian-origin strains, such as G17P[17] and G18P[17], have also been identified in bovine populations. These findings collectively support the occurrence of interspecies transmission from pigs and birds to cattle. Additionally, bovine genotypes VP7 G1, G2, G3, G4, G12, and VP4 P[6] have been found in human RVA cases, highlighting the zoonotic potential of these viruses [6].

It is noteworthy that reassortment was observed in the only successfully isolated strain (0205HG), suggesting the possibility of genetic exchange between bovine and heterologous rotavirus strains. However, given that only one isolate was sequenced, this observation should be interpreted with caution. Broader sampling and additional successful isolations are needed to assess the true frequency and epidemiological significance of such reassortment events. Due to the frequent and close contact between cattle and humans, the likelihood of interspecies transmission of BRV is elevated. Investigating the presence of BRV in children suffering from diarrhea who have regular exposure to cattle could provide important insights into its potential impact on public health.

These findings highlight the risk of zoonotic transmission of RVA and reinforce the importance of continuous monitoring and investigation of RVA strains in both human and animal populations. This study contributes to our understanding of the genetic variation and evolutionary patterns of bovine RVA strains, offering valuable information that could support the development of targeted vaccines and effective strategies to prevent and manage RVA infections in cattle.

## 5. Conclusions

In this study, we successfully isolated and characterized the BRV strain 0205HG from diarrheic calves in Inner Mongolia, China. Genotypic analysis revealed that this strain possesses the constellation G6-P[1]-I2-R2-C2-M2-A3-N2-T6-E2-H3, indicating it is a reassortant comprising gene segments from bovine, human, and feline rotaviruses. Notably, the VP4 gene of strain 0205HG shared high similarity with that of human rotavirus, underscoring the potential risk of zoonotic transmission. These results contribute valuable knowledge regarding the genetic diversity, evolutionary dynamics, and cross-species transmission potential of bovine rotavirus A. To mitigate the risks associated with emerging reassortant strains such as G6P[1], we recommend strengthening routine surveillance and molecular monitoring programs in cattle farms. Furthermore, our results highlight the importance of considering emerging genotypes in the selection and updating of vaccine strains. Enhancing farm-level biosecurity measures, including improved sanitation, isolation of sick animals, and immunization of dams, may help reduce transmission risk. Continued One Health-based monitoring in both animal and human populations is essential to guide evidence-based vaccination and control strategies against rotavirus infections.

## Figures and Tables

**Table 1:** RVA segment genotypes and closest homologs in the GenBank database.

Gene	Genotype	Closest Strain	Nucleotide Sequence Identity (%)	Amino Acid Sequence Identity (%)	Host Origin	Accession Number
VP1	R2	RVA/Human-wt/HUN/BP1062/2004/G8P[14]	93.4%	99.36%	Human	FN665688
VP2	C2	RVA/Yak-tc/CHN/HY-1/2018/G6P[11]	96.13%	99.42%	Bovine	QDJ94338
VP3	M2	RVA/Cat-wt/ITA/BA222/2005/G3P[9]	90.51%	95.21%	Feline	GU827408
VP4	P[1]	RVA/Human-tc/NGA/HMG035/1999/G8P[1]	91.80%	90.65%	Human	LC119096
VP6	I2	RVA/Cow-tc/USA/NCDV/1967/G9P[16]	99.63%	98.89%	Bovine	DQ870496
VP7	G6	RVA/Cow-wt/ARG/B3035_B_BA/2007/G6P[5]	96.62%	96.72%	Bovine	OM643804
NSP1	A3	RVA/Cow-tc/THA/A44/1989/G10P[11]	94.48%	94.97%	Bovine	LC133575.1
NSP2	N2	RVA/Cow-tc/JPN/AzuK-7/2007/G10P[11]	95.49%	96.85%	Bovine	LC553635
NSP3	T6	RVA/Cow-tc/USA/B223/1983/G10P[11]	98.84%	99.42%	Bovine	LC133555
NSP4	E2	RVA/Cow-tc/JPN/BRV106/1983/G6P[1]	99.55%	98.63%	Bovine	AB748604.1
NSP5	H3	RVA/Human-wt/USA/3000015004/2014/G6P[14]	97.82%	95.92%	Human	OP680466.1

**Table 2:** Genotype constellations of a representative 0205HG strain in comparison with other RVA strains of humans and various animal species.

Strain	G	P	I	R	C	M	A	N	T	E	H
0205HG	6	1	2	2	2	2	3	2	6	2	3
RVA/Human-tc/USA/DS-1/1976/G2P1B[4]	2	4	2	2	2	2	2	2	2	2	2
RVA/Human-tc/JPN/AU-1/1982/G3P3[9]	3	9	3	3	3	3	3	3	3	3	3
RVA/Cow-tc/ARG/B3700/2008/G6P[11]	6	11	2	5	2	2	3	2	6	12	3
RVA/Hu-wt/SVN/SI-R56/07/2007/G6P[11]	6	11	2	2	2	2	13	2	6	2	3
RVA/Cow-wt/IRL/CIT-A21/2006/G6P[11]	6	11	2	2	2	2	3	2	6	2	3
RVA/Cow-wt/SVN/SI-B17/2004/G6P[11]	6	11	2	2	2	2	3	2	6	2	3
RVA/Rabbit-tc/NLD/K1130027/2011/G6P[11]	6	11	2	2	2	2	13	2	6	2	3
RVA/Cow-wt/ZAF/MRC-456/2009/G6P[11]	6	11	2	2	2	2	13	2	6	2	3
RVA/Cow-wt/ZAF/MRC-3005/2009/G6P[11]	6	11	2	2	2	2	13	2	6	2	3
RVA/Cow-tc/UK/GBR/1973/G6P[5]	6	5	2	2	2	2	3	2	7	2	3
RVA/Cow-tc/ARG/B3035/2007/G6P[5]	6	5	2	5	2	2	3	2	6	12	3
RVA/Cow-tc/JPN/OH-4/1982/G6P[5]	6	5	2	2	2	2	13	2	6	2	3
RVA/Cow-wt/ZAF/1603/2007/G6P[5]	6	5	2	2	2	2	3	2	6	2	3
RVA/Cow-tc/FRN/RF/1982/G6P[1]	6	1	2	2	2	2	3	2	6	2	3
RVA/Cow-tc/USA/WC3/1981/G6P[1]	6	1	2	2	2	2	3	2	6	2	3
RVA/Cow-tc/USA/NCDV/1967/G6P[1]	6	1	2	2	2	2	3	2	6	2	3
RVA/Goat-tc/UGA/BUW-14/2014/G6P[1]	6	1	2	2	2	2	11	2	6	2	3
RVA/Goat-tc/BGD/GO34/1999/G6P[1]	6	1	2	2	2	2	11	2	6	2	3
RVA/Human-wt/GHA/PML1965/2012/G6P[6]	6	6	2	2	2	2	2	2	2	2	2
RVA/Human-wt/BEL/B1711/2002/G6P[6]	6	6	2	2	2	2	2	2	2	2	2
RVA/Human-wt/GNB/MRC-DPRU5625/2011/G6P[6]	6	6	2	2	2	2	2	2	2	2	2
RVA/Human-wt/CMR/MA202/2011/G6P[6]	6	6	2	2	2	2	2	2	2	2	2
RVA/Human-wt/BGD/KH2288/2011/G6P[8]	6	8	2	2	2	2	11	2	6	2	3
RVA/Human-wt/JPN/KF17/2010/G6P[9]	6	9	2	2	2	2	3	2	3	3	3
RVA/Human-tc/ITA/PA169/1988/G6P[14]	6	14	2	2	2	2	3	2	6	2	3
RVA/Human-wt/HUN/Hun5/1997/G6P[14]	6	14	2	2	2	2	11	2	6	2	3
RVA/Human-wt/EGY/EGY3399/2004/G6P[14]	6	14	2	2	2	2	11	2	6	2	3
RVA/Human-wt/IND/N-1/2009/G6P[14]	6	14	2	2	2	2	11	2	6	2	3
RVA/Human-tc/AUS/MG6/1993/G6P[14]	6	14	2	2	2	2	11	2	6	2	3
RVA/Human-wt/BEL/B10925/1997/G6P[14]	6	14	2	2	2	2	3	2	6	2	3
RVA/Human-wt/HUN/BP1879/2003/G6P[14]	6	14	2	2	2	2	11	2	6	2	3
RVA/Camel–wt/SDN/MRC–DPRU447/2002/G8P[11]	8	11	2	2	2	2	18	2	6	2	1
RVA/Cow-tc/THA/A44/1989/G10P[11]	10	11	2	2	2	2	3	2	6	2	3
RVA/Cow-tc/USA/B223/1983/G10P[11]	10	11	2	2	2	2	13	2	6	2	3
RVA/Giraff e-wt/IRL/GirRV/2008/G10P[11]	10	11	2	2	2	2	3	2	6	2	3
RVA/Cow-tc/JPN/KK3/1983/G10P[11]	10	11	2	2	2	2	13	2	6	2	3
RVA/Human-wt/IND/N155/200 3/G10P[11]	10	11	2	2	2	2	1	1	1	2	3
RVA/Cow-tc/CHN/DQ-75/2008/G10P[11]	10	11	2	2	2	2	3	2	6	2	3
RVA/Cow-tc/ARG/B383/1998/G15P[11]	15	11	2	5	2	2	13	2	6	12	3
RVA/Human-tc/USA/DS-1/1976/G2P1B[4]	2	4	2	2	2	2	2	2	2	2	2
RVA/Human-re/GBR/Re/1987/G10P[14]	10	14	2	2	2	1	3	2	6	2	3
RVA/Human-wt/IND/BCH 5836/2015/G3P[4]	3	4	1	1	1	1	1	1	2	1	1
RVA/Cow-wt/ZAF/1604/2007/G8P[1]	8	1	2	2	2	2	3	2	6	2	3
RVA/Human-wt/IND/BCH 7221/2015/G3P[8]	3	8	1	1	1	1	1	1	1	1	1

## Data Availability

The original contributions presented in this study are included in the article/Supplementary Material. Further inquiries can be directed to the corresponding author.

## Supplementary Materials

The following supporting information can be downloaded at https://www.mdpi.com/article/10.3390/vetsci12080742/s1: Table S1: The sequences of the RV reference strains used in this study were retrieved from the National Center for Biotechnology Information nucleotide database (GenBank).
