# Supplementary material for: Molecular Surveillance and Whole Genomic Characterization of Bovine Rotavirus A G6P[1] Reveals Interspecies Reassortment with Human and Feline Strains in China
Source: Vet Sci. 2025 Aug 7;12(8):742. doi: 10.3390/vetsci12080742 (PMC12389768; doi:10.3390/vetsci12080742)
Supplement: Supplementary file 1 [file vetsci-12-00742-s001.zip › vetsci-3755473-supplementary.pdf]

Table S1. The sequences of the RV reference strains used in this study were retrieved from the National Center for Biotechnology Information nucleotide database (GenBank).

Strains name	Hosts	GenBank accession no.
RVA/Porcine-wt/THA/CP29/08/2008/G3P13	Porcine	AB779634.1
RVA/Simian-wt/USA/RRV/1975/G3P3	Simian	AY117048.1
RVA/Horese-tc/AUS/Cat97/1984/G3P3	Horse	EU708951.1
RVA/Cow-wt/ARG/B383/1998/G15P11	Bovine	FJ347117.1
RVA/Cat-wt/ITA/BA222/2005/G3P9	Feline	GU827412.1
RVA/Cow-wt/ARG/B2592_B_Co/2004/G6P[11]	Bovine	KC895859.1
RVA/Human-wt/CHN/E3239/2012/G3P[8]	Human	KF371884
RVA/Human-wt/CHN/L1621/2013/G3P8	Human	KF371941.1
RVA/Human-wt/CHN/L1450/2012/G3P8	Human	KF371950.1
RVA/Murine-wt/USA/EB-cc40/1932/G16P16	Murine	KJ477276.1
RVA/Porcine-tc/BRA/ROTA18/2013/GXPX	Porcine	KJ482261.1
RVA/Human-wt/USA/CNMC125/2011/G1P[8]	Human	KT920687.1
RVA/Cow-tc/THA/A5-13/1988/G8P[1]	Human	LC133527.1
RVA/Human-wt/IDN/SOEP033/2016/G3P8	Human	LC260277.1
RVA/Human-wt/IDN/SOEP156/2016/G3P6	Human	LC260285.1
RVA/Cat-tc/JPN/FRV384/1994/G3P[9]	Feline	LC328209.1
RVA/Cat-tc/JPN/FRV384/1994/G3P9	Feline	LC328245.1
RVA/Horese-wt/JPN/MK9/2019/G13P18	Horse	LC528247.1
RVA/Human-wt/CHN/Hu/JS2011/2011/G9P8	Human	MF580848.1
RVA/Human-wt/CHN/Hu/JS2011/2011/G9P8	Human	MF580911.1
RVA/Human-wt/CHN/Hu/JS2013/2013/G9P8	Human	MF580913.1
RVA/Porcine-w't/MOZ/MZ-MPT-192/2016/G9P13	Porcine	MT784837.1
RVA/Cow-wt/ZAF/MRC-DPRU456/2009/G6P11	Bovine	MW771179.1
RVA/Cow-wt/TUR/K063/2008/G10P11	Bovine	MZ848179.1
RVA/Porcine-wt/CHN/FJSH01/2021/G26P23	Porcine	ON093984.1
RVA/Porcine-w't/XXX/Gottfried/1994/GXPX	Porcine	U08431.1

EB-aa40/G16P[16]	Murine	KJ477222.1
RVA/Cow-wt/JPN/Tottori-SG/2013/G15P[14]	Bovine	AB853895.1
CJ31-6	Porcine	AB905214.1
RVA/Pig-wt/JPN/BU2/2014/G5P[7]	Porcine	AB924087.1
RVA/Pig-wt/JPN/BU8/2014/G4P[6]	Porcine	AB924103.1
RVA/Porcine-w't/JPNBU9/2014/G9P23	Porcine	AB924112.1
RVA/Cow-tc/USA/WC3/1981/G6P[5]	Bovine	ABV66076.1
RVA/Cow-tc/VEN/BRV033/1990/G6P6[1]	Bovine	ABV66077.1
F8P4	Porcine	AFR33134.1
DS-1	Human	AJ540227.1
RVA/Cow-wt/TUR/Amasya-2/2015/G8P[5]	Bovine	APZ86782.1
RVA/Simian-tc/USA/RRV/1975/G3P[3]	Simian	AY117048.1
RVA/Cow-tc/USA/B223/1983/G10P[11]	Bovine	BAV35166.1
RVA/Human-wt/AUS/CK20038/2008/G6P[4]	Human	CK20038
SA11-1A	Simian	DQ838634.1
SA11-30/1A (SA11g4Oagentg5delC[30/1A])	Simian	DQ838644.1
SA11-30/1A	Simian	DQ838649.1
RVA/Cow-tc/USA/NCDV/1967/G9P16	Bovine	DQ870496.1
RVA/Human-tc/JPN/YO/1977/G3P1	Human	DQ870500.1
RVA/Human-wt/BEL/B3458/2003/G9P8	Human	DQ870504.1
RVA/Human-tc/AUS/MG6/1993/G6P14	Human	EF554097.1
RVA/Human-wt/HUN/Hun5/1997/G6P[14]	Human	EF554104.1
RVA/Human-wt/BEL/B10925/1997/G6P[14]	Human	EF554115.1
RVA/Human-tc/ITA/PA169/1988/G6P[14]	Human	EF554127.1
RVA/Human-wt/ITA/111-05-27/2005/G6P[14]	Human	EF554137.1
RVA/Human-wt/ITA/111-05-27/2005/G6P[14]	Human	EF554138.1
RVA/Sheep-tc/ESP/OVR762/2002/G8P[14]	Lamb	EF554148.1
RVA/Sheep-tc/ESP/OVR762/2002/G8P[14]	Lamb	EF554149.1
RVA/Sheep-tc/ESP/OVR762/2002/G8P[14]	Lamb	EF554150.1

RVA/Sheep-tc/ESP/OVR762/2002/G8P[14]	Lamb	EF554152.1
RVA/Sheep-tc/ESP/OVR762/2002/G8P[14]	Lamb	EF554154.1
RVA/Sheep-tc/ESP/OVR762/2002/G8P[14]	Lamb	EF554155.1
RVA/Sheep-tc/ESP/OVR762/2002/G8P[14]	Lamb	EF554156.1
RVA/Sheep-tc/ESP/OVR762/2002/G8P[14]	Lamb	EF554157.1
RVA/Sheep-tc/ESP/OVR762/2002/G8P[14]	Lamb	EF554158.1
RVA/Cow-tc/VEN/BRV033/1990/G6P6[1]	Bovine	EF560612.1
RVA/Human-tc/IND/69 M/1980/G8P4[10]	Human	EF576937.1
RVA/Simian-tc/USA/RRV/1975/G3P[3]	Simian	EF583007.1
RVA/Simian-tc/USA/RRV/1975/G3P[3]	Simian	EF583008.1
RVA/Human-tc/USA/WI61/1983/G9P1A[8]	Human	EF672619.1
RVA/Pig-tc/VEN/A131/1988/G3P9[7]	porcine	EF990688.1
RVA/Human-wt/BEL/B3458/2003/G9P[8]	Human	EF990711.1
RVA/Simian-tc/USA/RRV/1975/G3P[3]	Simian	EU636927.1
RVA/Simian-tc/USA/RRV/1975/G3P[3]	Simian	EU636931.1
RVA/Simian-tc/USA/RRV/1975/G3P[3]	Simian	EU636932.1
Cat97	Feline	EU708945.1
Cat97	Feline	EU708946.1
Cat97	Feline	EU708947.1
Cat97	Feline	EU708949.1
Cat97	Feline	EU708950.1
Cat97	Feline	EU708951.1
Cat97	Feline	EU708952.1
Cat97	Feline	EU708953.1
Cat97	Feline	EU708954.1
Cat97	Feline	EU708955.1
Lamb-NT	Lamb	FJ031019.1
Lamb-NT	Lamb	FJ031020.1
Lamb-NT	Lamb	FJ031021.1

Lamb-NT	Lamb	FJ031022.1
Lamb-NT	Lamb	FJ031023.1
Lamb-NT	Lamb	FJ031024.1
Lamb-NT	Lamb	FJ031025.1
Lamb-NT	Lamb	FJ031026.1
Lamb-NT	Lamb	FJ031028.1
RVA/Cow-wt/ARG/B383/1998/G15P[11]	Bovine	FJ347114.1
RVA/Cow-wt/ARG/B383/1998/G15P[11]	Bovine	FJ347116.1
RVA/Cow-wt/ARG/B383/1998/G15P[11]	Bovine	FJ347118.1
RVA/Cow-wt/ARG/B383/1998/G15P[11]	Bovine	FJ347119.1
RVA/Human-wt/HUN/BP1062/2004/G8P[14]	Human	FN665688.1
RVA/Human-wt/HUN/BP1062/2004/G8P[14]	Bovine	FN665689.1
GER1H-09	Human	GQ414543.1
RVA/Murine-tc/USA/ETD_822/2007/G3P10	Murine	GQ479952.1
ETD_822	Murine	GQ479953.1
ETD_822	Murine	GQ479955.1
RVA/Cat-wt/ITA/BA222/2005/G3P[9]	Feline	GU827406.1
RVA/Cat-wt/ITA/BA222/2005/G3P[9]	Feline	GU827407.1
RVA/Cat-wt/ITA/BA222/2005/G3P[9]	Feline	GU827408.1
RVA/Cat-wt/ITA/BA222/2005/G3P[9]	Feline	GU827410.1
RVA/Cat-wt/ITA/BA222/2005/G3P[9]	Feline	GU827412.1
RVA/Cat-wt/ITA/BA222/2005/G3P[9]	Feline	GU827413.1
RVA/Cat-wt/ITA/BA222/2005/G3P[9]	Feline	GU827414.1
RVA/Cat-wt/ITA/BA222/2005/G3P[9]	Feline	GU827415.1
RVA/Cat-wt/ITA/BA222/2005/G3P[9]	Feline	GU827416.1
GO34	Lamb	GU937877.1
GO34	Lamb	GU937878.1
GO34	Lamb	GU937879.1
GO34	Lamb	GU937881.1

GO34	Lamb	GU937883.1
GO34	Lamb	GU937884.1
GO34	Lamb	GU937885.1
GO34	Lamb	GU937886.1
GO34	Lamb	GU937887.1
DS-1	Human	HQ650124.1
CC0812-1/2008	Lamb	HQ834200.1
CC0812-1/2008	Lamb	HQ834202.1
CC0812-1/2008	Lamb	HQ834203.1
CC0812-1/2008	Lamb	HQ834204.1
CC0812-1/2008	Lamb	HQ834205.1
CC0812-1/2008	Lamb	HQ834206.1
CC0812-1/2008	Lamb	HQ834207.1
RVA/Horse-tc/GBR/L338/1991/G13P[18]	Horse	JF712555.1
RVA/Horse-tc/GBR/L338/1991/G13P[18]	Horse	JF712556.1
RVA/Horse-tc/GBR/L338/1991/G13P[18]	Horse	JF712557.1
RVA/Horse-tc/GBR/L338/1991/G13P[18]	Horse	JF712558.1
RVA/Horse-tc/GBR/L338/1991/G13P18	Horse	JF712559.1
RVA/Horse-tc/GBR/L338/1991/G13P[18]	Horse	JF712560.1
RVA/Horse-tc/GBR/L338/1991/G13P[18]	Horse	JF712561.1
RVA/Horse-tc/GBR/L338/1991/G13P[18]	Horse	JF712562.1
RVA/Horse-tc/GBR/L338/1991/G13P[18]	Horse	JF712563.1
RVA/Horse-tc/GBR/L338/1991/G13P[18]	Horse	JF712564.1
RVA/Horse-tc/GBR/L338/1991/G13P[18]	Horse	JF712565.1
RVA/Cow-wt/ZAF/1603/2007/G6P[5]	Bovine	JN831209.1
RVA/Cow-wt/ZAF/1603/2007/G6P5	Bovine	JN831213.1
F8P4	Porcine	JN974795.2
LLR	Lamb	JQ013502.1
LLR	Lamb	JQ013503.1

LLR	Lamb	JQ013504.1
LLR	Lamb	JQ031145.1
LLR	Lamb	JQ031146.1
LLR	Lamb	JQ031147.1
LLR	Lamb	JQ031148.1
LLR	Lamb	JQ031150.1
LLR	Lamb	JQ031151.1
RVA/Horse-wt/ZAF/EqRV-SA1/2006/G14P[12]	Horse	JQ345490.1
RVA/Horse-wt/ZAF/EqRV-SA1/2006/G14P[12]	Horse	JQ345492.1
RVA/Horse-wt/ZAF/EqRV-SA1/2006/G14P[12]	Horse	JQ345493.2
RVA/Horse-wt/ZAF/EqRV-SA1/2006/G14P[12]	Horse	JQ345495.1
RVA/Horse-wt/ZAF/EqRV-SA1/2006/G14P[12]	Horse	JQ345499.1
RVA/Horse-wt/ARG/E3198/2008/G3P[3]	Horse	JX036368.1
RVA/Horse-wt/ARG/E3198/2008/G3P3	Horse	JX036369.1
RVA/Horse-wt/ARG/E3198/2008/G3P[3]	Horse	JX036371.1
RVA/Horse-wt/ARG/E3198/2008/G3P[3]	Horse	JX036373.1
RVA/Human-wt/ITA/AV21/2010/G9P8	Human	JX195067.1
RVA/Human-wt/ITA/JES11/2010/G9P8	Human	JX195089.1
RVA/Yak-tc/CHN/QH-1/2015/G6P[1]	Bovine	K638899.1
RVA/Cow-wt/ARG/B1186_B_ER/2000/G6P[5]	Bovine	KC895761.1
RVA/Cow-wt/ARG/B3553_D_BA/2008/G6P[5]	Bovine	KC895770.1
RVA/Cow-wt/ARG/B3035_B_BA/2007/G6P[5]	Bovine	KC895772.1
RVA/Cow-wt/ARG/B1541/2001/G6P[11]	Bovine	KC895794.1
RVA/Cow-wt/ARG/B3679_D_BA/2008/G10P[11]	Bovine	KC895796.1
RVA/Cow-wt/ARG/4181_D_BA/2003/G10P[5]	Bovine	KC895799.1
RVA/Cow-wt/ARG/B3110_D_BA/2006/G10P[11]	Bovine	KC895806.1
RVA/Cow-wt/ARG/791_BA/1999/G10P[5]	Bovine	KC895808.1
RVA/Cow-wt/ARG/792_BA/1999/G10P[5]	Bovine	KC895810.1
RVA/Cow-wt/ARG/791_BA/1999/G10P[5]	Bovine	KC895826.1

RVA/Cow-wt/ARG/B2376_D_BA/2003/G10P[5]	Bovine	KC895831.1
RVA/Cow-wt/ARG/B611_BA/1999/G6P[11]	Bovine	KC895841.1
RVA/Cow-wt/ARG/B1988_BA/2002/G6P[11]	Bovine	KC895847.1
RVA/Cow-wt/ARG/B3700_D_BA/2008/G6P[11]	Bovine	KC895860.1
RVA/Human-wt/CHN/E2432/2010/G3P[8]	Human	KF371854.1
RVA/Human-wt/CHN/L1621/2013/G3P[8]	Human	KF371937.1
RVA/Human-wt/CHN/L1450/2012/G3P[8]	Human	KF371951.1
174-1	Porcine	KF500214.1
C-1	Porcine	KF500218.1
RVA/Human-wt/CHN/E2484/2011/G4P[8]	Human	KF726044.1
RVA/Human-wt/CHN/E2484/2011/G4P[8]	Human	KF726045.1
RVA/Human-wt/CHN/E2484/2011/G4P8	Human	KF726046.1
EB-A8/G16P[16]	Murine	KJ477106.1
RVA/Murine-wt/USA/EB-A8/1982/G16P16	Murine	KJ477109.1
EB-A8/G16P[16]	Murine	KJ477110.1
RVA/Murine-wt/USA/EB-A8/1982/G16P16	Murine	KJ477111.1
EB-A8/G16P[16]	Murine	KJ477112.1
EB-A8/G16P[16]	Murine	KJ477113.1
EB-B8/G16P[16]	Murine	KJ477116.1
EB-B8/G16P[16]	Murine	KJ477122.1
EB-B8/G16P[16]	Murine	KJ477123.1
EB-B8/G16P[16]	Murine	KJ477124.1
EB-B8/G16P[16]	Murine	KJ477125.1
EB-B8/G16P[16]	Murine	KJ477126.1
EB-E7/G16P[16]	Murine	KJ477194.1
EB-aa40/G16P[16]	Murine	KJ477215.1
EB-aa40/G16P[16]	Murine	KJ477216.1
RVA/Murine-wt/USA/EB-aa40/1982/G16P16	Murine	KJ477221.1
EB-aa40/G16P[16]	Murine	KJ477223.1

EB-cc40/G16P[16]	Murine	KJ477270.1
EB-cc40/G16P[16]	Murine	KJ477271.1
RVA/Murine-wt/USA/EB-cc40/1982/G16P16	Murine	KJ477274.1
EB-cc40/G16P[16]	Murine	KJ477275.1
EB-cc40/G16P[16]	Murine	KJ477277.1
EB-cc40/G16P[16]	Murine	KJ477278.1
EB-kk18-pl-4-3-1-1/G16P[16]	Murine	KJ477314.1
EB-kk18-pl-4-3-1-1/G16P[16]	Murine	KJ477315.1
RVA/Murine-wt/USA/EB-kk18-D1-4-3-1-1/1982/G16P16	Murine	KJ477318.1
EB-kk18-pl-4-3-1-1/G16P[16]	Murine	KJ477319.1
RVA/Murine-wt/USA/EB-kk18-p4-3-1-1/1982/G16P16	Murine	KJ477320.1
EB-kk18-pl-4-3-1-1/G16P[16]	Murine	KJ477321.1
EB-kk18-pl-4-3-1-1/G16P[16]	Murine	KJ477322.1
RVA/Porcine-w't/BRA/ROTA23/2013/GXPX	Porcine	KJ482266.1
RVA/Cow-wt/ZAF/MRC-DPRU3005/2009/G6P[5]	Bovine	KJ751923.1
RVA/Cow-wt/ZAF/MRC-DPRU3010/2009/G6P[5]	Bovine	KJ752062.1
RVA/Cow-wt/ZAF/MRC-DPRU3010/2009/G6P[5]	Bovine	KJ752064.1
RVC/Pig-wt/KOR/07-109-12/2007/G6PX	Porcine	KJ814473.1
RVC/Pig-wt/KOR/1027/2012/G7PX	Porcine	KJ814508.1
RVC/Pig-wt/CZE/P303/2011	Porcine	KM099263.1
RVC/Pig-wt/CZE/P44/2013	Porcine	KM099264.1
RVC/Pig-wt/CZE/P59/2013	Porcine	KM099271.1
RVA/Human-wt/TGO/MRCDPRU5123/2010/G9P[8]	Human	KP752519.1
RVC/Pig-wt/CZE/P21/2013	Porcine	KP776735.1
RVC/Pig-wt/CZE/P141/2010	Porcine	KP776736.1
RVA/Human-wt/MWI/OP354/1998/G4P[8]	Human	KP902534.1
RVA/Human-wt/USA/Wa/1974/G1P[8]	Human	KT694942.1
RVA/Human-wt/USA/Wa/1974/G1P[8]	Human	KT694944.1
RVA/Pig-wt/THA/CMP-001-12/2012/G5P[13]	Porcine	KT727244.1

RVA/Human-wt/USA/CNMC125/2011/G1P[8]	Human	KT920689.1
RVA/Human-wt/MWI/MW2-181_B/2000/G1P[8]	Human	KU714455.1
RVA/Pig-wt/TWN/4-1/2015/G9P19	Porcine	KU739963.1
RVA/Cow-wt/TUR/Amasya-2/2015/G8P[5]	Bovine	KX212870.1
RVA/Cow-wt/TUR/Amasya-2/2015/G8P[5]	Bovine	KX212876.1
RVA/Human-wt/UGA/MUL-13-204/2013/G8P[6]	Human	KX655451.1
RVA/Cow-wt/UGA/BUW-14-A035/2014/G12P[8]	Bovine	KX655528.1
RVA/Cow-wt/UGA/BUW-14-A035/2014/G12P8	Bovine	KX655537.1
RVA/Simian-wt/KNA/08979/2015/G5P[X]	Simian	KY053144.1
RVA/Simian-wt/KNA/08979/2015/G5P[X]	Simian	KY053145.1
RVA/Simian-wt/KNA/08979/2015/G5P[X]	Simian	KY053149.1
RVA/Simian-wt/KNA/08979/2015/G5P[X]	Simian	KY053150.1
RVA/Simian-wt/KNA/08979/2015/G5P[X]	Simian	KY053151.1
RVA/Simian-wt/KNA/08979/2015/G5P[X]	Simian	KY053152.1
RVA/Simian-wt/KNA/08979/2015/G5P[X]	Simian	KY053153.1
RVA/Pig-wt/UGA/BUW-14-A008/2014/G12P8	Bovine	KY055419.1
12059	Human	KY113343.1
HY-1	Bovine	KY865389.1
RVA/Human-tc/JPN/AU109/1994/G8P[4]	Human	LC065020.1
RVA/Human-tc/NGA/HMG035/1999/G8P[1]	Human	LC119096.1
RVA/Cow-tc/NGA/NGRBg8/1998/G8P[1]	Bovine	LC119104.1
RVA/Cow-tc/NGA/NGRBg8/1998/G8P[1]	Bovine	LC119105.1
RVA/Cow-tc/NGA/NGRBg8/1998/G8P[1]	Bovine	LC119106.1
RVA/Cow-tc/NGA/NGRBg8/1998/G8P[1]	Bovine	LC119108.1
RVA/Cow-tc/NGA/NGRBg8/1998/G8P[1]	Bovine	LC119110.1
RVA/Cow-tc/NGA/NGRBg8/1998/G8P[1]	Bovine	LC119111.1
RVA/Cow-tc/NGA/NGRBg8/1998/G8P[1]	Bovine	LC119112.1
RVA/Cow-tc/NGA/NGRBg8/1998/G8P[1]	Bovine	LC119113.1
RVA/Cow-tc/NGA/NGRBg8/1998/G8P[1]	Bovine	LC119114.1

RVC/Pig-wt/Tochigi-1-1/2015/G9P[4]	Porcine	LC122593.1
RVC/Pig-wt/Tottori-KT01/2015/G13P[4]	Porcine	LC122611.1
RVC/Pig-wt/Tottori-KT01/2015/G13P[4]	Porcine	LC122614.1
RVA/Cow-tc/THA/A5-13/1988/G8P[1]	Bovine	LC133525.1
RVA/Cow-tc/JPN/KK3/1983/G10P[11]	Bovine	LC133558.1
RVA/Cow-tc/JPN/KK3/1983/G10P11	Bovine	LC133562.1
RVA/Cow-tc/THA/A44/1989/G10P[11]	Bovine	LC133570.1
RVA/Cow-tc/THA/A44/1989/G10P11	Bovine	LC133575.1
RVA/Cow-tc/THA/A44/1989/G10P[11]	Bovine	LC133577.1
RVA/Human-wt/IDN/SOEP075/2016/G3P[8]	Human	LC260213.1
RVA/Human-wt/IDN/SOEP128/2016/G3P[6]	Human	LC260215.1
RVA/Human-wt/IDN/SOEP152/2016/G3P[8]	Human	LC260218.1
RVA/Human-wt/IDN/SOEP156/2016/G3P[6]	Human	LC260219.1
RVA/Human-wt/IDN/SOEP003/2015/G3P[8]	Human	LC260220.1
RVA/Human-wt/IDN/SOEP018/2015/G3P[8]	Human	LC260221.1
RVA/Human-wt/IDN/SOEP033/2015/G3P[8]	Human	LC260222.1
RVA/Human-wt/IDN/SOEP044/2015/G3P[8]	Human	LC260223.1
RVA/Human-wt/IDN/SOEP075/2016/G3P[8]	Human	LC260224.1
RVA/Human-wt/IDN/SOEP101/2016/G3P[8]	Human	LC260225.1
RVA/Human-wt/IDN/SOEP128/2016/G3P[6]	Human	LC260226.1
RVA/Human-wt/IDN/SOEP137/2016/G3P[8]	Human	LC260227.1
RVA/Human-wt/IDN/SOEP003/2015/G3P8	Human	LC260231.1
RVA/Human-wt/IDN/SOEP018/2015/G3P8	Human	LC260232.1
RVA/Human-wt/IDN/SOEP033/2015/G3P8	Human	LC260233.1
RVA/Human-wt/IDN/SOEP144/2016/G3P8	Human	LC260239.1
RVA/Human-wt/IDN/SOEP156/2016/G3P6	Human	LC260241.1
RVA/Human-wt/IDN/SOEP003/2015/G3P[8]	Human	LC260242.1
RVA/Human-wt/IDN/SOEP018/2015/G3P[8]	Human	LC260243.1
RVA/Human-wt/IDN/SOEP033/2015/G3P[8]	Human	LC260244.1

RVA/Human-wt/IDN/SOEP044/2015/G3P[8]	Human	LC260245.1
RVA/Human-wt/IDN/SOEP101/2016/G3P[8]	Human	LC260247.1
RVA/Human-wt/IDN/SOEP144/2016/G3P[8]	Human	LC260250.1
RVA/Human-wt/IDN/SOEP003/2015/G3P[8]	Human	LC260253.1
RVA/Human-wt/IDN/SOEP033/2015/G3P[8]	Human	LC260255.1
RVA/Human-wt/IDN/SOEP044/2015/G3P[9]	Human	LC260256.1
RVA/Human-wt/IDN/SOEP101/2016/G3P[8]	Human	LC260258.1
RVA/Human-wt/IDN/SOEP152/2016/G3P[8]	Human	LC260262.1
RVA/Human-wt/IDN/SOEP003/2016/G3P8	Human	LC260275.1
RVA/Human-wt/IDN/SOEP018/2016/G3P8	Human	LC260276.1
RVA/Human-wt/IDN/SOEP044/2016/G3P8	Human	LC260278.1
RVA/Human-wt/IDN/SOEP075/2016/G3P6	Human	LC260279.1
RVA/Human-wt/IDN/SOEP101/2016/G3P8	Human	LC260280.1
RVA/Human-wt/IDN/SOEP128/2016/G3P6	Human	LC260281.1
RVA/Human-wt/IDN/SOEP137/2016/G3P8	Human	LC260282.1
RVA/Human-wt/IDN/SOEP144/2016/G3P8	Human	LC260283.1
RVA/Human-wt/IDN/SOEP152/2016/G3P8	Human	LC260284.1
RVA/Human-wt/IDN/SOEP101/2016/G3P[8]	Human	LC260291.1
RVA/Human-wt/IDN/SOEP128/2016/G3P[6]	Human	LC260292.1
RVA/Human-wt/IDN/SOEP137/2016/G3P[8]	Human	LC260293.1
RVA/Human-wt/IDN/SOEP144/2016/G3P[8]	Human	LC260294.1
RVA/Human-wt/IDN/SOEP152/2016/G3P[8]	Human	LC260295.1
RVA/Human-wt/IDN/SOEP156/2016/G3P[6]	Human	LC260296.1
RVA/Human-wt/IDN/SOEP003/2015/G3P[8]	Human	LC260297.1
RVA/Human-wt/IDN/SOEP018/2015/G3P[8]	Human	LC260298.1
RVA/Human-wt/IDN/SOEP033/2015/G3P[8]	Human	LC260299.1
RVA/Human-wt/IDN/SOEP044/2015/G3P[8]	Human	LC260300.1
RVA/Human-wt/IDN/SOEP075/2016/G3P[8]	Human	LC260301.1
RVA/Human-wt/IDN/SOEP128/2016/G3P[6]	Human	LC260303.1

RVA/Human-wt/IDN/SOEP156/2016/G3P[6]	Human	LC260307.1
RVA/Cat-tc/JPN/FRV384/1994/G3P[9]	Feline	LC328215.1
RVA/Cat-tc/JPN/FRV348/1994/G3P[3]	Feline	LC328219.1
RVA/Cat-tc/JPN/FRV317/1994/G3P[9]	Feline	LC328220.1
RVA/Cat-tc/JPN/FRV384/1994/G3P9	Feline	LC328221.1
RVA/Cat-tc/JPN/FRV348/1994/G3P[3]	Feline	LC328225.1
RVA/Cat-tc/JPN/FRV317/1994/G3P[9]	Feline	LC328226.1
RVA/Cat-tc/JPN/FRV348/1994/G3P[3]	Feline	LC328231.1
RVA/Cat-tc/JPN/FRV317/1994/G3P[9]	Feline	LC328232.1
RVA/Cat-tc/JPN/FRV348/1994/G3P[3]	Feline	LC328237.1
RVA/Cat-tc/JPN/FRV317/1994/G3P[9]	Feline	LC328238.1
RVA/Cat-tc/JPN/FRV348/1994/G3P[3]	Feline	LC328243.1
RVA/Cat-tc/JPN/FRV317/1994/G3P[9]	Feline	LC328244.1
RVA/Cat-tc/JPN/FRV348/1994/G3P[3]	Feline	LC328249.1
RVA/Cat-tc/JPN/FRV317/1994/G3P[9]	Feline	LC328250.1
RVA/Cat-tc/JPN/FRV384/1994/G3P[9]	Feline	LC328251.1
RVA/Cat-tc/JPN/FRV348/1994/G3P[3]	Feline	LC328255.1
RVA/Cat-tc/JPN/FRV317/1994/G3P[9]	Feline	LC328256.1
RVA/Cat-tc/JPN/FRV384/1994/G3P[9]	Feline	LC328257.1
RVA/Cat-tc/JPN/FRV317/1994/G3P[9]	Feline	LC328262.1
RVA/Cat-tc/JPN/FRV348/1994/G3P[3]	Feline	LC328267.1
RVA/Cat-tc/JPN/FRV317/1994/G3P[9]	Feline	LC328268.1
RVA/Horse-tc/JPN/MK9/2019/G13P[18]	Horse	LC528247.1
RVA/Horse-tc/JPN/MK9/2019/G13P[18]	Horse	LC528248.1
RVA/Horse-tc/JPN/MK9/2019/G13P[18]	Horse	LC528249.1
RVA/Horse-tc/JPN/MK9/2019/G13P[18]	Horse	LC528250.1
RVA/Horse-tc/JPN/MK9/2019/G13P[18]	Horse	LC528251.1
RVA/Horse-tc/JPN/MK9/2019/G13P[18]	Horse	LC528255.1
RVA/Horse-tc/JPN/MK9/2019/G13P[18]	Horse	LC528257.1

RVA/Cow-tc/JPN/GB1-76/2006/G10P[11]	Bovine	LC553591.1
RVA/Cow-tc/JPN/GB12-22/2007/G8P11	Bovine	LC553601.1
RVA/Cow-tc/JPN/GB14-45/2007/G6P11	Bovine	LC553612.1
RVA/Cow-tc/JPN/AzuK-7/2007/G10P[11]	Bovine	LC553635.1
RVC/Cow-wt/JPN/Ishi-Mi21/2021/G3P10	Bovine	LC622281.1
RVC/Cow-wt/JPN/Ishi-Mi21/2021/G3P10	Bovine	LC622286.1
RVC/Cow-wt/JPN/Ishi-Mi39/2021/G3P10	Bovine	LC622292.1
RVC/Pig-wt/JPN/Ishi-Im1/2015/G1P4PX	Porcine	LC622301.1
RVC/Pig-wt/JPN/Ishi-Im9/2016/G1PX	Porcine	LC622314.1
RVC/Pig-wt/JPN/Ishi-Ka6/2016/G1P4	Porcine	LC622327.1
RVC/Pig-wt/JPN/HgYa3/2016/GXPX	Porcine	LC622389.1
RVA/Human-wt/CHN/Hu/JS2010/2010/G9P8	Human	MF580847.1
RVA/Human-wt/CHN/Hu/JS2012/2012/G9P8	Human	MF580849.1
RVA/Human-wt/CHN/Hu/JS2013/2013/G9P8	Human	MF580850.1
RVA/Human-wt/CHN/Hu/JS2014/2014/G9P8	Human	MF580851.1
RVA/Human-wt/CHN/Hu/JS2015/2015/G9P8	Human	MF580852.1
RVA/Human-wt/CHN/Hu/JS2016/2016/G9P8	Human	MF580853.1
Hu/JS2010	Human	MF580854.1
Hu/JS2013	Human	MF580856.1
Hu/JS2014	Human	MF580857.1
Hu/JS2015	Human	MF580858.1
Hu/JS2016	Human	MF580859.1
Hu/JS2011	Human	MF580860.1
Hu/JS2011	Human	MF580876.1
Hu/JS2012	Human	MF580877.1
Hu/JS2013	Human	MF580878.1
Hu/JS2014	Human	MF580879.1
Hu/JS2015	Human	MF580880.1
Hu/JS2016	Human	MF580881.1

Hu/JS2010	Human	MF580882.1
Hu/JS2010	Human	MF580896.1
Hu/JS2011	Human	MF580897.1
Hu/JS2012	Human	MF580898.1
Hu/JS2013	Human	MF580899.1
Hu/JS2014	Human	MF580900.1
Hu/JS2015	Human	MF580901.1
Hu/JS2016	Human	MF580902.1
Hu/JS2011	Human	MF580904.1
Hu/JS2012	Human	MF580905.1
Hu/JS2013	Human	MF580906.1
Hu/JS2014	Human	MF580907.1
Hu/JS2015	Human	MF580908.1
Hu/JS2016	Human	MF580909.1
RVA/Human-wt/CHN/Hu/JS2010/2010/G9P8	Human	MF580910.1
RVA/Human-wt/CHN/Hu/JS2014/2014/G9P8	Human	MF580914.1
RVA/Human-wt/CHN/Hu/JS2015/2015/G9P8	Human	MF580915.1
RVA/Porcine-tc/KOR/K71/2006/G5P[7]	Porcine	MF940437.1
RVA/Porcine-tc/KOR/K71/2006/G5P7	Porcine	MF940442.1
RVA/Porcine-tc/KOR/K71/2006/G5P[7]	Porcine	MF940457.1
RVA/Porcine-tc/KOR/PRG942/2006/G9P23	Porcine	MF940507.1
RVA/Porcine-tc/KOR/174-1/2006/G8P[7]	Porcine	MF940555
RVI/Cat-wt/FRA/MG/2017/GXP	Feline	MG779487.1
RVA/Porcine-w't/THA/CMP-011-09/2009/G4P6	Porcine	MG781058.1
RVA/Pig-wt/CHN/SCLSHL-2-3/2017/G9P[23]	Porcine	MH137272.1
MN.9.65	Porcine	MH267269.1
RVC/Pig-wt/USA/OK.5.68/2008	Porcine	MH282893.1
BatRVA322/Taphozous mauritianus/KEN/Kwale/2015	Bat	MH285828.1
BatRVA322/Taphozous mauritianus/KEN/Kwale/2015	Bat	MH285829.1

BatRVA322/Taphozous mauritianus/KEN/Kwale/2015	Bat	MH285832.1
BatRVA322/Taphozous mauritianus/KEN/Kwale/2015	Bat	MH285833.1
BatRVA322/Taphozous mauritianus/KEN/Kwale/2015	Bat	MH285834.1
BatRVA322/Taphozous mauritianus/KEN/Kwale/2015	Bat	MH285836.1
OK.5.68	Porcine	MH308716.1
RVA/Porcine-w't/USA/OK5.68b/2008/GXPX	Porcine	MH308721.1
RVA/Porcine-w't/CHN/SCLSHL-2-3/2017/G9P23	Porcine	MH898993.1
RVA/Yak-tc/CHN/HY-1/2018/G6P[11]	Bovine	MK250424.1
RVA/Yak-tc/CHN/HY-1/2018/G6P[11]	Bovine	MK250425.1
RVA/Yak-tc/CHN/HY-1/2018/G6P[11]	Bovine	MK250426.1
RVA/Yak-tc/CHN/HY-1/2018/G6P[11]	Bovine	MK250428.1
RVA/Yak-tc/CHN/HY-1/2018/G6P[11]	Bovine	MK250430.1
RVA/Yak-tc/CHN/HY-1/2018/G6P[11]	Bovine	MK250431.1
RVA/Yak-tc/CHN/HY-1/2018/G6P[11]	Bovine	MK250432.1
RVA/Yak-tc/CHN/QH-1/2015/G6P[1]	Bovine	MK638870.1
RVA/Yak-tc/CHN/HY-1/2018/G6P[11]	Bovine	MK638876.1
RVA/Yak-tc/CHN/HY-1/2018/G6P[11]	Bovine	MK638879.1
RVA/Yak-tc/CHN/HY-1/2018/G6P[11]	Bovine	MK638880.1
RVA/Cow-wt/URY/LVMS3031/2016/G6P[11]	Bovine	MN649721.1
RVA/Cow-wt/URY/LVMS3073/2016/G6P[11]	Bovine	MN649728.1
RVA/Cow-wt/URY/LVMS3690/2017/G6P[11]	Bovine	MN649730.1
RVA/Cow-wt/URY/LVMS781/2015/G6P[5]	Bovine	MN649752.1
RVA/Cow-wt/URY/LVMS1788/2016/GxP[11]	Bovine	MN649753.1
RVA/Cow-wt/URY/LVMS3206/2016/GxP[11]	Bovine	MN649761.1
RVA/Cow-wt/CHN/XJX2/2018/G10P[X]	Bovine	MN937506.1
RVA/Cow-tc/CHN/SDC1/2018/G6P[1]	Human	MN937523.1
K-98	Lamb	MT501452.1
K-98	Lamb	MT501453.1
K-98	Lamb	MT501454.1

K-98	Lamb	MT501456.1
K-98	Lamb	MT501458.1
K-98	Lamb	MT501459.1
K-98	Lamb	MT501460.1
K-98	Lamb	MT501461.1
K-98	Lamb	MT501462.1
RVA/Pig-wt/MOZ/MZ-MPT-192/2016/G9P[13]	Porcine	MT784790.1
RVA/Pig-wt/MOZ/MZ-MPT-192/2016/G9P[13]	Porcine	MT784797.1
RVA/Pig-wt/MOZ/MZ-MPT-192/2016/G9P[13]	Porcine	MT784805.1
RVA/Pig-wt/MOZ/MZ-MPT-195/2016/G9P[13]	Porcine	MT784810.1
RVA/Pig-wt/MOZ/MZ-MPT-192/2016/G9P[13]	Porcine	MT784813.1
RVA/Pig-wt/MOZ/MZ-MPT-192/2016/G9P[13]	Porcine	MT784821.1
RVA/Pig-wt/MOZ/MZ-MPT-192/2016/G9P[13]	Porcine	MT784837.1
RVA/Pig-wt/MOZ/MZ-MPT-195/2016/G9P[13]	Porcine	MT784842.1
RVA/Pig-wt/MOZ/MZ-MPT-192/2016/G9P[13]	Porcine	MT784845.1
RVA/Pig-wt/MOZ/MZ-MPT-192/2016/G9P[13]	Porcine	MT784853.1
RVA/Pig-wt/MOZ/MZ-MPT-192/2016/G9P[13]	Porcine	MT784869.1
RVA/Human-wt/USA/Wa/1974/G1P8	Human	MT796883.1
BatRVH/Bat-wt/CH/Myo_dau2/2019	Bat	MT815950.1
BatRVH/Bat-wt/CH/Myo_dau2/2019	Bat	MT815953.1
BatRVH/Bat-wt/CH/Myo_dau2/2019	Bat	MT815957.1
BatRVH/Bat-wt/CH/Myo_dau3/2019	Bat	MT815963.1
BatRVH/Bat-wt/CH/Myo_dau2/2019	Bat	MT815965.1
NJ2012	Porcine	MT874983.1
NJ2012	Porcine	MT874984.1
NJ2012	Porcine	MT874985.1
NJ2012	Porcine	MT874987.1
NJ2012	Porcine	MT874988.1
NJ2012	Porcine	MT874989.1

NJ2012	Porcine	MT874990.1
NJ2012	Porcine	MT874992.1
NJ2012	Porcine	MT874993.1
RVA/Cow-wt/ZAF/Bov4/2003/G6P[5]	Bovine	MW771133.1
RVA/Cow-wt/ZAF/Bov7/2003/G10P[11]	Bovine	MW771140.1
RVA/Cow-wt/ZAF/Bov7/2003/G10P1	Bovine	MW771142.1
RVA/Cow-wt/ZAF/Bov7/2003/G10P[11]	Bovine	MW771144.1
RVA/Cow-wt/MOZ/MPT-93/2016/G10P11	Bovine	MW771153.1
RVA/Cow-wt/MOZ/MPT-93/2016/G10P[11]	Bovine	MW771154.1
RVA/Cow-wt/MOZ/MPT-307/2016/G10P11	Bovine	MW771158.1
RVA/Cow-wt/MOZ/MPT-93/2016/G10P[11]	Bovine	MW771159.1
RVA/Cow-wt/MOZ/MPT-307/2016/G10P[11]	Bovine	MW771165.1
RVA/Cow-wt/MOZ/MPT-307/2016/G10P[11]	Bovine	MW771169.1
RVA/Cow-wt/ZAF/MRC-DPRU457/2009/G10P11	Bovine	MW771186.1
RVA/Cow-wt/ZAF/MRC-DPRU457/2009/G10P[11]	Bovine	MW771187.1
RVA/Cow-wt/ZAF/MRC-DPRU457/2009/G10P11	Bovine	MW771190.1
RVA/Cow-wt/ZAF/MRC-DPRU457/2009/G10P[11]	Bovine	MW771192.1
RVA/Cow-wt/ZAF/MRC-DPRU3005/2009/G6P11	Bovine	MW771197.1
RVA/Cow-wt/ZAF/MRC-DPRU3005/2009/G6P[11]	Bovine	MW771202.1
RVA/Porcine-wt/CHN/HB-1/2019/GXPX	Porcine	MZ165494.1
RVA/Porcine-wt/CHN/HB-7/2019/GXPX	Porcine	MZ165495.1
RVA/Cow-wt/JPN/Sun-9/2008/G8P11	Bovine	MZ314041.1
RVA/Cow-wt/CHN/LN12/2018/G6P[1]	Bovine	N937497.1
BatRVA/KEN/BATp39/Rousettus aegyptiacus/2015	Bat	NC_040406.1
BatRVA/KEN/BATp39/Rousettus aegyptiacus/2015	Bat	NC_040408.1
BatRVA/KEN/BATp39/Rousettus aegyptiacus/2015	Bat	NC_040409.1
RVA/Bat-wt/KEN/BATP39/2015/G36P51	Bat	NC_040410.1
BatRVA/KEN/BATp39/Rousettus aegyptiacus/2015	Bat	NC_040412.1
BatRVA/KEN/BATp39/Rousettus aegyptiacus/2015	Bat	NC_040413.1

BatRVA/KEN/BATp39/Rousettus aegyptiacus/2015	Bat	NC_040414.1
RVA/Horse-wt/IND/ERV6/2017/G3P[3]	Horse	OK651081.1
RVA/Horse-wt/IND/ERV6/2017/G3P[3]	Horse	OK651093.1
RVA/Horse-wt/IND/ERV6/2017/G3P[3]	Horse	OK651097.1
RVA/Horse-wt/IND/ERV6/2017/G3P3	Horse	OK651098.1
RVA/Horse-wt/IND/ERV6/2017/G3P[3]	Horse	OK651113.1
RVA/Cow-wt/CHN/SCMY-1/2021/GXPX	Bovine	ON012969.1
RVA/Cow-tc/China/SCMY1/2021	Bovine	ON012970.1
RVA/Cow-tc/China/SCMY2/2021	Bovine	ON012981.1
RVA/Porcine-tc/CHN/FJSH01/2021/G26P23	Porcine	ON093975.1
RVA/Pig/China/FJSH01/2021/G26P[23]	Porcine	ON093977.1
RVA/Pig/China/FJSH01/2021/G26P[23]	Porcine	ON093985.1
RVA/Lamb-wt/CHN/LLR/1985/G10P12	Lamb	OQ603392.1
RVA/Lamb-wt/CHN/LLR/1985/G10P12	Lamb	OQ603393.1
LLR	Lamb	OQ603394.1
LLR	Lamb	OQ603395.1
RVA/Murine-wt/USA/EW/1994/GXPX	Murine	U08428.1
RVA/Cow-wt/ZAF/Bov1/2009/G6P[5]	Bovine	UQQ66538.1
RVA/Cow-wt/ZAF/Bov4/2003/G6P[5]	Bovine	UQQ66539.1
RVA/Cow-wt/TUR/K56/2006/G6P[11]	Bovine	UYH99833.1
